# Identifying factors associated with depression among men living with HIV/AIDS and undergoing antiretroviral therapy: a cross-sectional study in Heilongjiang, China

**DOI:** 10.1186/s12955-018-1020-x

**Published:** 2018-09-19

**Authors:** Huan Liu, Miaomiao Zhao, Jiaojiao Ren, Xinye Qi, Hong Sun, Lemeng Qu, Cunling Yan, Tong Zheng, Qunhong Wu, Yu Cui

**Affiliations:** 1 Department of Social Medicine, School of Public Health, Harbin Medical University, 157 Baojian Road, Nangang District, Harbin, Heilongjiang China; 2 Department of Health Management, School of Humanities and Management, Jinzhou Medical University, Jinzhou, China; 3 Department of Pharmacy, Third Affiliated Hospital of Harbin Medical University (Tumor Hospital of Harbin Medical University), Harbin, China

**Keywords:** Antiretroviral therapy, Depression, HIV/AIDS, Influencing factors, Male

## Abstract

**Background:**

Depression is common among people living with HIV/AIDS; however, studies focusing on the depression of men living with HIV/AIDS are limited. Therefore, we examined the prevalence of depression and its associated factors among men living with HIV/AIDS in China.

**Methods:**

A cross-sectional questionnaire survey was conducted in Harbin, China between March and August in 2013. Two-hundred twenty participants completed the Burns Depression Checklist, the Berger HIV Stigma, and the SPIEGEL questionnaire. We also investigated demographics, family support, hostility, and the antiretroviral therapy side effects of men living with HIV/AIDS.

**Results:**

More than 40% of respondents had depressive symptoms and worry about the health was the major symptom of depression (40.9%). The logistic regression model indicated that bad sleep quality (OR = 3.452), hostility (OR = 1.120), perceived discrimination (OR = 1.110), and antiretroviral therapy side effects (OR = 1.083) were positively associated with depression. Family support (OR = 0.860) was negatively associated with depression for men living with HIV/AIDS. Demographic variables, HIV infection route, disease duration, and CD4+ cell count had no significant associations with depression.

**Conclusion:**

Although China’s work of national HIV prevention and treatment has made much progress during the past several years, the prevalence of depression among men living with patients with HIV/AIDS is still prominent. The strongest factor associated with depression among men living with HIV/AIDS was sleep quality. Future studies should explore the effects of interventions for depression among PLWHA.

## Background

Acquired immune deficiency syndrome (AIDS), which is caused by the human immunodeficiency virus (HIV), is a global health crisis. In 2017, 36.9 million people had HIV and 940,000 people were dying of AIDS [1]. In China, more than 820,000 people were living with HIV/AIDS as of June 2018 [2]. AIDS and HIV are costly from both an economic and human suffering standpoint.

Globally, it is estimated that 4.4% of the global population suffered from depressive disorder in 2015 [3]. Depression is a major cause of disability as measured by Years Lived with Disability; it contributed to almost 7.5% of all disabilities [4]. Among people living with HIV/AIDS (PLWHA), depression is the most frequently observed psychiatric disorder [5]. In Nigeria, Adewuya et al. found that the rate of psychiatric disorders in individuals with HIV was 59.1% compared to 19.5% without HIV infection [6]. According to Bengtson and colleagues, who examined 31,000 HIV-infected adults in the United States, nearly 47% of the participants had depression problems [7]. A cross-country, multicenter, cross-sectional epidemiological study of HIV+ patients conducted in Western Europe and Canada found that the positive rate of screening for depression was 15.7% [8]. Moreover, the proportion of PLWHA with depressive symptoms is particularly high in developing countries. For example, India is home to the third-largest number of people living with HIV in the world [9]. Many scholars have found that the prevalence of depression is high among HIV-infected Indian individuals, with a prevalence ranging from 25 to 67.3% [10–13]. In Brazil, recent preliminary estimates of depression in HIV-infected Brazilians vary from 21.8–37% [14–17]. A high incidence of depression among PLWHA is also common in China. Sun and colleagues reported that 73.1% of PLWHA had depression [18], which is much higher than the rate in the general population in China (15.1–22.5%) [19].

Among PLWHA, not only are depressive symptoms very common, but the impact of them is also multifaceted. On the one hand, depression can lead to dire consequences for PLWHA. PLWHA with depression are approximately 2 times more likely to have poor medication compliance than those without depression [20]. Depression symptoms can also damage the immune system, thus negatively affecting health [21]. On the other hand, depression can also lead to dire consequences for society. PLWHA with depression have a higher risk of transmitting AIDS through drug abuse and unsafe sexual behavior [22]. In summary, depression has a profound impact on PLWHA and society.

In previous studies, several factors have been found to be associated with depression such as education [23], income [24], and CD4 counts [25]. Furthermore, compared to the general population, HIV-infected persons have a higher prevalence of insomnia [26, 27]. Lee et al. found that only 30% of PLWHA were good sleepers [28]. Moreover, some studies have suggested that insomnia may be related to depression among PLWHA in some countries [26, 27]. However, the relationship between insomnia and depression still needs to be confirmed among PLWHA in China. A national-level policy, known as “Four Frees and One Care” (free screening test, free treatment of poor AIDS patients, free schooling for orphans of AIDS patients, free counseling and antiretroviral therapy for pregnant women with HIV, and social assistance for HIV patients) began in 2003 and is now widely available in China [29]. Although this program substantially reduced the economic burden on patients and increased treatment accessibility, but many patients have experienced ART side effects. Phillips et al. found that 97% of participants reported experiencing at least one side effect after ART initiation [30, 31]. Side effects of long-term medication are bound to affect mental health among PLWHA. Chen and colleagues found that depression can be a pernicious consequence of ART side effects in PLWHA [31].

Previous studies showed that PLWHA have been severely discriminated against not only by the general population [32], but also by healthcare providers [33]. Surprisingly, some PLWHA have even suffered discrimination from their families [31]. Self-stigma or perceived discrimination from others will directly impact the psychological well-being of PLWHA in the future [34]. Several studies have indicated that stigma contributes to depression in PLWHA [35–37]. In addition, among the many aspects of psychological health, family support is undoubtedly a key factor. Amiya et al.’s findings highlighted an important role for family support in determining experiences of depression among PLWHA [38]. Besides, hostility has been a neglected psychological problem among PLWHA. Some researchers have found that, among PLWHA, an extremely hostile mentality was not uncommon [39]. The association between hostility and depression was mentioned as early as the 1990s [40]. However, no data is available on the effect of hostility on depression among PLWHA.

To date, many studies about depression in PLWHA have focused on women, while men living with HIV/AIDS (MLWHA) have been largely neglected. Raso et al. assessed the associations between depression and physical fitness and function in MLWHA and the role that sexual satisfaction plays in these associations [41]. Esposito et al. found that up to 1 in 5 men living with HIV in Vietnam experiences clinically significant depressive symptoms and the prevalence of depression may increase as a function of HIV-related life stressors [42]. MLWHA account for the vast majority of PLWHA in China, and 71.4% of PLWHA were male in 2011 [43]. Further, a previous review found that the prevalence of depression among MLWHA in China was very high (37.9–71.8%) [44]. From a gender perspective, MLWHA also have unique depression-related coping patterns and mental health needs. Vosvick and colleagues showed that in men living with HIV, greater use of distraction, blame, and expression and lower use of positive growth were related to greater depression [45]. Moreover, Vosvick et al. also found that depression was associated with symptoms and higher use of blame in women living with HIV [45]. Daughtry et al. explored the gender differences in depression-related coping patterns and found that women’s unique coping includes a willingness to seek help, while coping for men entails individual resolution of difficulties [46]. For these reasons, it is important to understand the prevalence of depression in MLWHA, their psychological needs, as well as the relationship between depression and influencing factors in them.

This study aimed to examine depression and its potential influencing factors in MLWHA in China. Related factors were compared in depressed and non-depressed MLWHA patients, risk and protective factors were identified and discussed. The findings might provide evidence and support for the future’s effective prevention and treatment of depression in PLWHA.

## Methods

### Data collection

A cross-sectional survey of MLWHA patients who received antiretroviral therapy (ART) was conducted in the first affiliated hospital of Harbin medical university between March and August 2013. Patients who participated in this study were included based on the following criteria: aged 18 years or older, male living with AIDS/HIV, having received ART, and able to read and write Chinese. Patients meeting the criteria were informed by their physicians about the study and provided consent to participate. Trained researchers conducted the survey. All participants were informed that the study was voluntary and confidential. After provided written informed consent by participants, interviews were conducted. The database used in this study contained identification numbers to protect the privacy of participants. The study was approved by the Ethics Review Board of Harbin Medical University. In total, 227 questionnaires were completed and 220 (97%) were valid and used for data analysis.

### Variables

A structured questionnaire was designed to obtain demographic and social economic characteristics, depression, perceived discrimination, subjective sleep quality evaluation, ART side effects, and family support and hostility.

#### Dependent variable

The dependent (outcome) variable was depressive symptoms of MLWHA. To assess depression, we used the Burns Depression Checklist (BDC). The BDC consisted of 15 dimensions of depression. Participants were asked to rate how much each of the depressive symptoms bothered them in the last several days [47]. The patients rated the frequency of each potential symptom as 0 (“*none*”) to 3 (“*a lot*”). The BDC yields a total depression score ranging from 0 to 45. BDC scores are divided into several categories: minimal or no depression (0–4), borderline depression (5–10), mild depression (11–20), moderate depression (21–30), and severe depression (31–45). The BDC has good internal consistency, strong content validity, excellent concurrent validity, and well-established discriminant validity [48]. We evaluated the reliability of the BDC with the current sample and found that the scale possessed excellent reliability (alpha coefficient = 0.96). This coefficient is consistent with the internal consistency estimate for the BDC that was reported by Burns in a sample of clinically depressed individuals (i.e., .90) [49]. Higher total scores indicate more severe depression [50]. In this paper, a BDC score > 11 was used to identify men with depressive symptoms [51].

#### Independent variable

Demographic and socio-economic characteristics included age, marital status, number of children, education background, household monthly income per capita, disease duration, infection route, and CD4+ cell count.

The Berger HIV Stigma scale was developed by Barbara Berger and colleagues [52]. We revised it to measure the level of perceived discrimination in MLWHA. The 40 items of the Berger HIV Stigma scale were rated on a 4-point Likert scale (*strongly disagree, disagree, agree, strongly agree*) [53]. Higher scores reflect greater perceived HIV stigma [54]. We found in a previous study that participants were very resistant to this scale, which greatly affected the quality of the survey [55]. Considering participants’ possible exclusion of this scale and to not affect the quality of the survey, we used a simplified Berger HIV Stigma scale [55]. This new scale included 20 items in four dimensions: personalized stigma, disclosure concerns, negative self-image, and concern with public attitudes. The scale had high internal consistency (Cronbach’s α = 0.90) in the present study, which was consistent with prior research of PLWHA (Cronbach’s α = 0.90) [55].

We used the 6-item SPIEGEL questionnaire to measure sleep quality. The SPIEGEL questionnaire is a widely used international scale developed by R. Spiegel in 1984 [56–59]. Respondents report the time it takes to fall asleep (0 = *within thirty minutes*, 1 = *within one hour*, 3 = *within two hours*, 5 = *over two hours*, and 7 = *over four hours*), sleep duration (0 = *eight hours*, 1 = *six hours*, 3 *= four hours*, 5 = *two hours*, and 7 = *less than two hours*), number of nocturnal waking (0 = *zero times*, 1 = *once*, 3 = *twice*, 5 = *three times*, 7 = *four times*), sleep depth (0 = *ten points*, 1 = *eight points*, 3 = *six points*, 5 = *four points*, 7 = *two points*), frequency of nighttime dreaming (0 = *never*, 1 = *sometimes*, 3 = *often*, 5 = *usually*, 7 = *always*), and wake-up feeling (0 = *very good*, 1 = *good*, 3 = *fair*, 5 = *bad*, 7 = *very bad*). Higher total scores indicate worse sleep quality, and poor sleep quality was defined as a score between 18 and 42 [60]. The SPIEGEL questionnaire showed acceptable internal consistency in this sample (Cronbach’s α = 0.75).

Family support was measured using the family support dimension of a quality of life scale designed for Chinese people living with HIV/AIDS [61]. Participants were asked about their degree of family support with the following questions: “Can you obtain family care and support? Can you get help when you need help? Do you feel loved and cared for? Do you have close people to talk to?” Items were scored on a 5-point scale ranging from 1 (“*none of the time*”) to 5 (“*all the time*”). Higher scores indicate more family support for MLWHA. Cronbach’s α of this scale was 0.87, which indicated high internal consistency and was consistent with a past sample of PLWHA (Cronbach’s α = 0.89) [61].

We used the hostility dimension of a quality of life scale designed for Chinese PLWHA to estimate the respondent’s hostility trend; this scale has acceptable internal consistency (Cronbach’s α = 0.78) [61]. Moreover, the scale had high internal consistency (Cronbach’s α = 0.92) in the present study population. Participants were asked about their hostility using the following questions: “Do you give up on yourself? Do you feel like you hate the outside world? Do you want to kill the person who infected you? Do you think your illness is caused by society? Do you feel abandoned by society? Do you have the urge to retaliate against society?” Six statements regarding the frequency and severity of hostility in MLWHA were rated on a scale from 1 (*none of the time*) to 5 (*all the time*). Higher scores represented more hostility in MLWHA.

We used a self-designed scale to evaluate the degree of ART side effects in MLWHA. The scale comprised 14 items, and participants were asked to choose 1 answer for each statement (0 = *asymptomatic*, 1 = *symptoms, but insignificant*, 2 = *some trouble*, 3 = *very troubled*, and 4 = *extremely troubled*). Respondents reported their side effects, including appetite changes, nausea and vomiting, difficulty sleeping, abdominal pain, dry skin, rash, numbness in the limbs, pain in the limbs, fatigue, body shape changes, hair loss, vision changes, headache, and dreaminess. Total scores ranged 0–56, with higher scores indicating a higher level of ART side effects. Cronbach’s α for the ART side effects scale was 0.88.

### Statistical analyses

After the data cleaning process, incomplete or anomalous data (*n* = 17) were excluded, leaving a final sample of 220 participants. Descriptive statistics were calculated, including frequencies and percentages for binary/categorical variables, means and standard deviations for normally distributed continuous variables. Median and interquartile ranges were also presented for continuous variables that were not normally distributed. Univariate analysis was performed using Pearson’s chi-squared test, *t*-tests, or Wilcoxon’s rank sum test, and the multivariate logistic regression was used to examine associations between depression and its associated factors. All data were analyzed using SPSS 21.0, and *p* < .05 was considered significant.

## Results

### Participants’ characteristics

Participants’ general characteristics are shown in Table 1. Of the total sample (*N* = 220) most were aged 41 years or older (35%); unmarried, divorced, or widowed (72.3%); had no children (77.7%); and were educated at or beyond the undergraduate level (31.4%). More than half (54.1%) had the disease no more than three years. About 71% of the respondents had homosexual partners. Through the univariate analysis, we found that household monthly income per capita, dyssomnia, family support, hostility, higher ART side effects, and perceived discrimination were significantly associated with depression (*p* < .05).

**Table 1:** Participants’ characteristics per whether they have depression

Variable	Total	Depression		*p*-value^d^
	*N* = 220 (%)	Yes *n* = 98 (%)	No *n* = 122 (%)	
Age (years)^a^				.848
19–29	69 (31.4)	32 (32.7)	37 (30.3)	
30–40	74 (33.6)	31 (31.6)	43 (35.2)	
≥ 41	77 (35.0)	35 (35.7)	42 (34.4)	
Marital status^a^				.650
Married or cohabitating	61 (27.7)	29 (29.6)	32 (26.2)	
Unmarried, divorce, or widowed	159 (72.3)	69 (70.4)	90 (73.8)	
Have children^a^				.746
Yes	49 (22.3)	23 (23.5)	26 (21.3)	
No	171 (77.7)	75 (76.5)	96 (78.7)	
Education background^a^				.393
Junior high school and below	50 (22.7)	25 (25.5)	25 (20.5)	
High school/secondary school	50 (22.7)	23 (23.5)	27 (22.1)	
College	51(23.2)	25 (25.5)	26 (21.3)	
Undergraduate and above	69(31.4)	25 (25.5)	44 (36.1)	
Household monthly income per capita^a^				.026
3000 yuan and below	168(76.4)	82 (83.7)	86 (70.5)	
More than 3000 yuan	52(23.6)	16 (16.3)	36 (29.5)	
Disease duration (years)^a^				.363
< 2	38(17.3)	19 (19.4)	19 (15.6)	
2 to < 3	81(36.8)	35 (35.7)	46 (37.7)	
3 to < 4	34(15.5)	15 (15.3)	19 (15.6)	
4 to < 5	25(11.4)	7 (7.1)	18 (14.8)	
5 or more	42(19.1)	22 (22.4)	20 (16.4)	
Infection routes^a^				.301
Homosexual sex	156(70.9)	66 (67.3)	90 (73.8)	
Non-homosexual sex	64(29.1)	32 (32.7)	32 (26.2)	
CD4+ cell count^a^				.547
≤ 200	47(21.4)	25 (25.5)	22 (18.0)	
200–350	72(32.7)	32 (32.7)	40 (32.8)	
350–500	65(29.5)	27 (27.6)	38 (31.1)	
> 500	36(16.4)	14 (14.3)	22 (18.0)	
Sleep quality^a^				.004
Poor	23(10.5)	17 (17.3)	6 (4.9)	
Good	197(89.5)	81 (82.7)	116 (95.1)	
Family support^b^	16(12.25–19.75)	14 (11–16)	17 (14–20)	< .001
Hostility^b^	7(6–10)	9 (6–12)	6 (6–7)	< .001
ART side effects^b^	5(1–11)	8 (4–15)	3 (0–7)	< .001
Perceived discrimination^c^	52.81 ± 9.47	56.96 ± 7.79	49.48 ± 9.42	.022

^a^Data were expressed as no. (%); ^b^Data were expressed as median (P_25_–P_75_); ^c^Data were expressed as mean ± standard deviation; ^d^*p*-value was calculated using the t-test, chi-square test, and Wilcoxon’s rank-sum test where applicable

### The basic symptoms of depression

Table 2 shows the symptoms of depression among participants, which we identified as anyone who had symptoms with scores greater than 0 (“*none*”) for each item. Worrying about the health was the major symptom associated with depression (40.9%), followed by feeling discouraged (25%). Meanwhile, sadness (23.2%) was also a key symptom and 14.5% participants experienced irritability and frustration. In addition, 12.7% participants lost interest in life.

**Table 2:** Depression symptoms and rank ordering

Items	*N*	%	Rank
Worrying about the health	90	40.9	1
Feel discouraged	55	25	2
Sadness	51	23.2	3
Low self-esteem	49	22.3	4
Loss of libido	47	21.4	5
Indecisiveness	46	20.9	6
Inferiority	45	20.5	7
Guilt	40	18.2	8
Sleep changes	39	17.7	9
Poor self-image	38	17.3	10
Loss of motivation	35	15.9	11
Appetite changes	35	15.9	12
Suicidal impulses	35	15.9	13
Irritability and frustration	32	14.5	14
Loss of interest in life	28	12.7	15

### Sleep problems and other factors

Table 3 shows the sleep problems among participants. We found that 52% of MLWHA with depression could not fall sleep within 30 min, 73.5% of respondents with depression had more than one nocturnal waking. In addition, 20.4% of participants with depression felt bad when they awoke.

**Table 3:** Depression and sleep problems

Variable	Depression		*p*-value
	Yes *n* = 98 (%)	No *n* = 122 (%)	
Sleep latency (hours)			.005
≤ 0.5	47 (48.0)	83 (68.0)	
0.5 < to 1	30 (30.6)	28 (23.0)	
1<	21 (21.4)	11 (9.0)	
Sleep duration (hours)			.020
6 <	47 (48.0)	78 (63.9)	
≤ 6	51 (52.0)	44 (36.1)	
Number of nocturnal waking			.001
0	26 (26.5)	55 (45.1)	
1–2	53 (54.8)	59 (48.4)	
2<	8 (8.2)	19 (15.6)	
Sleep depth			.027
Good	59 (60.2)	91 (32.0)	
Fair	27 (27.6)	26 (26.5)	
Bad	11 (11.2)	5 (5.1)	
Nighttime dream			.002
Never	43 (43.9)	77 (63.1)	
Sometimes	33 (33.7)	36 (29.5)	
Always	22 (22.4)	9 (7.4)	
The feeling of waking up			< .001
Good	35 (35.7)	79 (64.8)	
Fair	43 (43.9)	36 (29.5)	
Bad	20 (20.4)	7 (5.7)	

Furthermore, we found that the ART side effects mainly include feeling tired (48%), dreaminess (33.7%), nausea, and vomiting (32.7%). In addition, we found that among hostile symptoms, self-abandonment, hate for the outside world, a feeling that they want to kill the person who infected them, attribution of the cause of the disease to society, and feeling abandoned by society were associated with depression (*p* < .05).

### The factors associated with depression per the logistic regression model

Independent variables that were significant predictors of depression in the chi-square tests, Wilcoxon’s rank-sum test, or t-tests were entered into the logistic regression analysis model. As shown in Table 4, sleep quality was the most significant influencing factor of depression among MLWHA. Compared with the respondents who had good sleep quality, respondents with poor sleep quality were 3.452 times (95% CI = 1.028–11.589) more likely to suffer from depression. Patients who had hostility were 1.12 times (95% CI = 1.046–1.199) more likely to suffer from depression. Other significant risk factors included perceived discrimination (OR = 1.11, 95% CI = 1.064–1.157) and ART side effects (OR = 1.083, 95% CI = 1.028–1.142). And family support can reduce the odds of depression in MLWHA (OR = 0.86, 95% CI = 0.789–0.937).

**Table 4:** The logistic regression analysis of factors associated with depression

Variable	β	Standard error	Wald	*p*-value	Odds ratio (95% confidence interval)
Sleep quality	1.239	0.618	4.019	.045	3.452 (1.028–11.589)
ART side effects	0.080	0.027	8.840	.003	1.083 (1.028–1.142)
Perceived discrimination	0.104	0.021	23.763	< .001	1.110 (1.064–1.157)
Family support	−0.151	0.044	11.773	< .001	0.860 (0.789–0.937)
Hostility	0.113	0.035	10.652	.001	1.120 (1.046–1.199)
Household monthly income per capita	−0.388	0.406	0.913	.339	0.678 (0.306–1.503)

## Discussion

The prevalence of depression of MLWHA was high in Heilongjiang: more than 40% of respondents had depressive symptoms and the rate was far higher than the general population in China [62]. Such an elevated level of depressive symptoms has also been found in studies in India [63]. Despite differences in data collection methods, it is evident that depression is very common in MLWHA. Through this study, we found significant influential factors of MLWHA, which can help doctors to understand the depression in MLWHA in greater depth.

In our study, 17.3% of participants with depressive symptoms reported poor sleep quality, and 4.9% of participants with non-depressive symptoms reported poor sleep quality. Multivariate logistic regression showed that the strongest risk factor associated with depression among MLWHA was sleep quality. This finding was consistent with other studies [21, 27, 64] and exemplified that bad sleep quality was a major factor in depression. However, compared with previous literature, this study highlighted the prominent role of sleep quality in the impact of depression among MLWHA. We also found that sleep latency, sleep duration, number of nocturnal waking, sleep depth, nighttime dream. and the feeling of waking up were significantly associated with depression among MLWHA. Given the bidirectional relationship between sleep and depression, management of one condition may improve the other. For example, Ford and Kamerow found that treating depression might improve sleep quality, and the treatment of sleep disturbances may decrease the incidence of depression [65]. Therefore, we recommend the implementation of a sleep quality assessment mechanism and timely interventions for patients with HIV/AIDS and depression.

Our results showed that hostility was the second highest contributory factor that influenced depression, and this positive relationship between hostility and depression in MLWHA is a novel finding in this study. In particular, we found that the major symptoms of hostility in depressive MLWHA were the appearance of self-abandonment, hate for the outside world, a desire to kill the person who infected them, the attribution of the cause of disease to society, and feeling abandoned by society. Hostile individuals display increased psychosocial vulnerability and experience more distress due to frequent interpersonal conflict, which results in social alienation and estrangement [66–68]. Furthermore, it can increase the psychological stress of patients and the risk of having depression. It was estimated that there are approximately about 550,000 MLWHA in China in 2011 [43]. If they have long-term hostility, it is bound to affect mental health and cause radical behavior for some, which may have grave consequences for society.

In this study, we found a positive correlation between perceived discrimination and depression. In 2011, an investigation of the prevalence of discrimination against HIV/AIDS patients in nine countries in Asia indicated that patients in each country reported a prominent level of internalized discrimination [69]. Su et al. found that perceived discrimination was associated with depression, and perceived stress mediates the relationship between perceived discrimination and depression [70]. Therefore, perceived discrimination is a psychological burden that may be associated with the occurrence of depression, and the elimination of discrimination requires active interventions at different levels. On one hand, the government and department of health should strengthen publicity and education about AIDS to deepen society’s understanding; on the other hand, patients should also learn to manage stress and actively seek mental health services.

The emergence of ART transformed HIV from a terminal illness to a chronic disease and resulted in significant decreases in HIV-related morbidity and mortality [71, 72]. However, ART side effects have led to psychological problems such as depression [31]. In this study, we found that ART side effects can increase the likelihood of depression in MLWHA. Moreover, we realized that worry about the health was a key symptom for depression. ART side effects undoubtedly increase patients’ concerns about their health. Therefore, in the treatment process of HIV/AIDS, doctors should pay attention to ART side effects and reduce the treatment of secondary injuries. Currently, there are many ways to alleviate ART side effects. In clinical practice, this often involves changing patients’ medication time, medication program, or the introduction of related non-prescription auxiliary drugs according to the side effects. Meanwhile, in China, an increasing number of patients have tried to improve their immunity and treat AIDS through traditional Chinese medicine [73]. In addition, Chen’s study emphasized that a manual of self-management for ART side effects should be provided.

Family support is crucial for patients with HIV/AIDS. It provides warmth and care and decreases the stress faced by patients [63]. However, only 57% of patients had a good relationship with their family, whereas the remainder considered them to be indifferent, hostile, or unsupportive [74]. Very few studies have investigated the impact of family support on depression in patients with HIV; however, it has been proposed that poor family support is associated with an increased incidence of depression in PLWHA [75]. We showed that family support was the most vital protective factor contributing to depression. Participants who reported lower family support were more likely to experience depressive symptoms. Consequently, communication with families is a key means to reduce the incidence of depression of patients. Although, some studies confirmed that MLWHA have a higher level of familial support than do women [76, 77], it is undeniable that PLWHA require more familial support over the duration of the disease.

Moreover, the demographic variables, HIV infection route, disease duration, and CD4+ cell count had no significant associations with depression. Many scholars have reached the same conclusion [78, 79], and they have studied the cause of depressive symptoms in AIDS patients from a clinical perspective. Treisman and colleagues’ study suggested that depression might be the result of the neurotropic effects of the virus on the subcortical brain areas in some people living with HIV/AIDS [80]. Through this study we can see that, in addition to the AIDS virus itself, patients’ physical, psychological, and social factors are caused by depression-related factors.

Many previous studies have confirmed that women living with HIV/AIDS are more likely than MLWHA to experience depression [7, 78, 81]. However, many studies identify fewer men with depression because they are less likely to ask for help [82]. In addition, previous research has found that women seek social support in stressful situations more often than men [46]. Men attributed their depression to failure to provide for their family and loss of social status [82]. Therefore, MLWHA need more help and support from society and family. At the same time, we also noticed that male patients with depression may adopt an antisocial personality in coping with depression [83]. Our research also confirmed that MLWHA have a certain degree of hostility, and that hostility is negatively related to depression. Therefore, paying attention to the mental health of MLWHA is not only a public health issue, but may maintain social health and safety.

MLWHA require more family support to cope with depressive symptoms. The side effects of ART have been noted, and MLWHA require guidance to dispel their concerns and fears. In addition, MLWHA’s emotions need to be identified and mediated in a timely manner. In China, policies such as the 5-Year Action Plans and Four Frees and One Care have mainly focused on physical health care and not on the psychological health of PLWHA [18]. Therefore, we have several suggestions for healthcare professionals and the government: (1) the diagnosis of depression in PLWHA should be promoted and popularized; (2) healthcare professionals should focus on psychological treatment based on gender differences among PLWHA; (3) Prompt diagnosis and treatment of sleep disturbances are advocated; (4) healthcare professionals should carefully inquire about ART side effects and the government should consider the implementation of a manual of self-management for ART side effects; (5) the government and the community should undertake charitable activities related to AIDS, and educate the general population about AIDS and the mental health problems of PLWHA; and (6) the government should not only focus on the physical health of PLWHA, but also adopt relevant policies to protect their psychological health, such as free psychological counseling and treatment.

Several limitations of this study should be acknowledged. First, as it is a cross-sectional study, we cannot establish causal relationships based on the results. Second, information on the exposure and outcome was obtained via a self-reported questionnaire that potentially constituted a reporting bias; however, it would most likely be a non-differential bias. Third, owing to limited resources, this survey was conducted only in Heilongjiang province. In addition, some self-designed scales were used in this study, and some original scales were revised. Although we performed a reliability test for each scale, the reliability of the scales still requires further validation. In future studies, a larger sample size is needed to identify depressive factors in patients with HIV/AIDS in China. Although we found potential factors that were associated with our outcomes, further studies should focus on improving sleep quality, alleviating side effects, reducing hostility and perceived discrimination, and strengthening family support to observe whether these actions are effective at reducing depression in patients with HIV/AIDS.

## Conclusion

Over 40% of the respondents from Heilongjiang province had depressive symptoms in this study. The major finding of this study was that, among MLWHA, poor sleep quality, serious ART side effects, strong hostility, and perceived discrimination were positively associated with depression. However, having effective family support can reduce the likelihood of depressive symptoms in MLWHA. We thus recommend that the diagnosis of depression in MLWHA should be promoted and popularized. Although China has made much progress on HIV prevention and cure recently, the prevalence of depression among men living with patients with HIV/AIDS is still prominent. Therefore, future studies should explore the effects of interventions for depression among PLWHA.

## Abbreviations

*ART*: antiretroviral therapy
*BDC*: Burns Depression Checklist
*MLWHA*: men living with HIV/AIDS
*PLWHA*: people living with HIV/AIDS
*YLD*: Years Lived with Disability
